# Landscape of cancer genomics and precision oncology in Japan, South Korea, China, and Australia

**DOI:** 10.1016/j.lanwpc.2026.101981

**Published:** 2026-09-10

**Authors:** Hideaki Bando, Hirokazu Okayama, Yoon Jung Chang, Jinpu Yu, Sean M. Grimmond, Sun-Young Kong, Xin Hu, Nikolajs Zeps, Ji-Youn Han, Jong-Il Kim, Kyosuke Seguchi, Masataka Amisaki, Yasutoshi Sakamoto, Takao Fujisawa, Riu Yamashita, Kazuto Kato, Koji Kono, Amber Johns, Takayuki Yoshino

**Affiliations:** a Department of Gastrointestinal Oncology, National Cancer Center Hospital East, Kashiwa, 277-8577, Japan; b Department for the Promotion of Drug and Diagnostic Development, National Cancer Center Hospital East, Kashiwa, 277-8577, Japan; c Department of Gastrointestinal Tract Surgery, Fukushima Medical University School of Medicine, Fukushima, 960-1295, Japan; d National Cancer Control Institute, National Cancer Survivorship Center, National Cancer Center, Goyang, 10408, Republic of Korea; e Cancer Molecular Diagnostics Core, Tianjin Medical University Cancer Institute and Hospital, National Clinical Research Center for Cancer, Tianjin, 300060, China; f Collaborative Centre for Genomic Cancer Medicine, University of Melbourne, Parkville, Australia; g Peter MacCallum Cancer Centre, Melbourne, Australia; h Department of Laboratory Medicine, National Cancer Center, Goyang, 10408, Republic of Korea; i Precision Cancer Medicine Center, Fudan University Shanghai Cancer Center, Shanghai, 200032, China; j Monash University Faculty of Medicine, Nursing and Health Sciences, School of Public Health and Preventive Medicine, Melbourne, Australia; k School of Health, Sports and Biosciences at the University of East London, Docklands campus, United Kingdom; l Center for Lung Cancer, National Cancer Center, Goyang, 10408, Republic of Korea; m Genomic Medicine Institute Medical Research Center, Cancer Research Institute, and Department of Biochemistry & Molecular Biology, Seoul National University College of Medicine, Seoul, 03080, Republic of Korea; n Department of Hepatobiliary and Pancreatic Oncology, National Cancer Center Hospital East, Kashiwa, 277-8577, Japan; o Department of Head and Neck Medical Oncology, National Cancer Center Hospital East, Kashiwa, 277-8577, Japan; p Division of Translational Informatics, Exploratory Oncology Research & Clinical Trial Center, National Cancer Center, Kashiwa, 277-8577, Japan; q Graduate School of Medicine and Premium Research Institute for Human Metaverse Medicine (WPI-PRIMe), The University of Osaka, Suita, Osaka, 565-0871, Japan; r International Cancer Genome Consortium (ICGC ARGO), Scotland, United Kingdom; s Pankind: Pancreatic Cancer Australia, Sydney, Australia; t Global Center of Research Excellence on Advanced Medicine (G-CoRE), Keio University School of Medicine, Tokyo, Japan

**Keywords:** Precision oncology, Cancer genomics, Asia-Pacific, Molecular tumour board, Genomic profiling, Health policy

## Abstract

Precision oncology has expanded rapidly across the Asia–Pacific region, yet implementation remains heterogeneous because of differences in health-system organisation, reimbursement, molecular tumour board infrastructure, workforce capacity, and data integration. This structured narrative review with expert consultation compares precision oncology ecosystems in Japan, South Korea, China, and Australia to identify common challenges and implementation strategies across the patient pathway. Although each country has established distinctive genomic medicine programmes, major disparities persist in access to comprehensive genomic profiling, molecularly matched therapies, and longitudinal data infrastructure. Comparative analysis highlights that successful implementation depends not only on sequencing capacity but also on coordinated governance, reimbursement alignment, multidisciplinary decision-making, research integration, and equitable access. Particular attention is required for geographically underserved populations and patients with rare or paediatric cancers. Based on these findings, we propose seven prioritised policy recommendations to strengthen precision oncology implementation, emphasising regional collaboration, interoperable data systems, workforce development, and sustainable reimbursement strategies to improve equity and clinical impact across the Asia–Pacific region.

**Key messages**

• Precision oncology is technically mature across Japan, South Korea, China, and Australia, yet implementation remains structurally heterogeneous. Variation in patient access is driven primarily by differences in reimbursement design, regulatory alignment, workforce distribution, and data governance rather than by sequencing capacity alone.
• The principal determinants of real-world impact lie along the implementation pathway. Eligibility restrictions for comprehensive genomic profiling (CGP), variability in molecular tumour board (MTB) governance, and misalignment between genomic findings and reimbursed therapies collectively shape treatment-matching rates across the region.
• Research-integrated ecosystems function as critical accelerators of precision oncology. National genomic programs, basket trials, virtual MTBs, and regulatory-grade real-world data platforms demonstrate how genomic testing can be linked to therapeutic access, longitudinal outcomes tracking, and policy-relevant evidence generation.
• Data governance and interoperability are emerging strategic inflection points. Federated data infrastructures, harmonised standards, and secure cross-border analytic frameworks are essential for enabling learning health systems while respecting national privacy and sovereignty requirements.
• Equity remains a cross-cutting imperative. Persistent urban–rural disparities, out-of-pocket financial barriers, and limited access for paediatric, rare, and molecularly uncommon cancers risk widening health inequalities unless proactively addressed through coordinated national reform and structured regional collaboration.

## Introduction

Cancer remains a leading cause of morbidity and mortality across the Asia–Pacific region. According to estimates from the Global Cancer Observatory (GLOBOCAN) 2022, nearly 20 million new cancer cases occurred worldwide in 2022.^1^ Japan, South Korea, China, and Australia together account for a substantial proportion of this global burden. China alone contributes approximately 4.82 million new cancer cases and 2.57 million cancer deaths annually, representing nearly one quarter of global incident cases.^2^ Japan and South Korea face an increasing cancer burden driven primarily by population ageing,^3^ while Australia reports among the highest age-standardised incidence rates globally.^1^ Ageing populations further increase demand for sustainable and personalised cancer care.

Several prevalent cancers in these countries are already amenable to precision oncology. Lung cancer remains the leading cause of cancer-related mortality, while breast, colorectal, and prostate cancers are among the most commonly diagnosed malignancies.^1^ In East Asia, colorectal cancer constitutes a major public health challenge, and gastric cancer remains highly prevalent, with emerging biomarker-driven treatment strategies.^4^ Advances in molecular profiling have transformed management by enabling targeted therapies and immunotherapies guided by genomic alterations such as *EGFR* mutations, *ALK* fusions, *HER2* amplification, *BRAF* mutations, and mismatch repair deficiency.^5^^,^^6^ However, clinical impact depends on effective health-system implementation rather than technology alone.^7^, ^8^, ^9^

National responses to cancer genomics and precision oncology vary substantially, reflecting differences in health-system organisation, reimbursement, and policy priorities. All four countries operate universal health coverage, yet coverage for genomic testing and targeted therapies differs markedly. These systems represent distinct implementation archetypes. Japan has embedded cancer genomic medicine within a nationally coordinated framework under its universal health insurance system, supported by designated hospitals and mandatory expert review.^10^, ^11^, ^12^, ^13^, ^14^ South Korea has achieved rapid adoption of next-generation sequencing, although reimbursement for comprehensive genomic profiling remains limited.^15^ China has expanded genomic technologies through public and private sectors, with increasing harmonisation through regional initiatives and evolving insurance frameworks. In Australia, selected pathology and genetic tests are reimbursed under Medicare, but comprehensive genomic profiling remains limited outside research and selected programs. However, Australia is highly active in precision oncology research, integrating genomic testing into clinical trials and research-embedded national genomics infrastructures that link molecular data with longitudinal clinical outcomes.^16^^,^^17^ These approaches mirror global observations that policy alignment, reimbursement, and infrastructure readiness are key determinants of implementation.

Equity considerations are central to understanding real-world impact. Despite universal coverage frameworks, disparities in access to molecular testing persist. Geographic inequities affect access to testing, specialised pathology services, and molecular tumour boards. Financial barriers—particularly out-of-pocket payment for comprehensive genomic profiling—remain a challenge. In Japan, age-related and institutional disparities influence access,^10^ while in Australia, Indigenous populations and those in remote regions experience gaps in access to care. Rare cancers, paediatric malignancies, and patients with uncommon genomic alterations are disproportionately affected, raising concerns that precision oncology may exacerbate existing disparities.

Against this background, a comparative regional assessment is timely. Japan, South Korea, China, and Australia were selected because they represent four complementary implementation archetypes within the Asia–Pacific region: a nationally coordinated public model, a rapidly evolving reimbursement-constrained system, a large-scale innovation- and market-driven ecosystem, and a research-integrated translational framework. Together, these countries capture the diversity of precision oncology implementation pathways across the region. These countries represent interrelated precision oncology ecosystems, encompassing policy-driven models, innovation-led systems, large-scale commercial expansion, and research-integrated frameworks. Examining them together provides an opportunity to identify shared challenges and enabling factors shaping patient pathways, consistent with calls to move beyond technology-centric evaluations toward system-level assessments.^9^

The objective of this narrative review is to map the current landscape of cancer genomics and precision oncology across the region, focussing on cancer burden, policy responses, patient pathways, access to testing and therapies, data governance, workforce capacity, and equity. By synthesising epidemiological data, policy frameworks, and real-world implementation, this review aims to inform the Lancet Oncology Commission and highlight opportunities for regional collaboration to advance equitable and sustainable precision oncology. Table 1 provides a structured comparison of key system characteristics.

**Table 1:** Comparative summary of national precision oncology programs and implementation characteristics across Japan, South Korea, China, and Australia.

Key Domain	Japan	South Korea	China	Australia
**Health system & national strategy**	Universal health coverage with a nationally coordinated cancer genomic medicine programme led by MHLW	Universal health coverage with rapid NGS adoption through a national health insurance pilot program alongside individual hospital-based MTB activities	Universal basic medical insurance with rapid expansion of cancer genomics driven by public hospitals and a large commercial diagnostics sector; national initiatives are increasingly harmonising standards and integration.	Universal health coverage
**Positioning of CGP in patient pathway**	Reimbursed CGP mainly restricted to patients after exhaustion of standard therapies	NGS capacity expanded through tertiary hospitals and private laboratories; limited reimbursement means many CGP tests are paid out-of-pocket	CGP widely available, especially in urban centres, but largely self-funded and heterogeneously integrated into routine care.	Restricted to research setting
**Testing infrastructure & quality assurance**	Centralised, quality-controlled national network using approved assays with mandatory standards	Institution-accreditation model requiring certification for NGS-based testing; considerable heterogeneity in panel design, gene coverage, and reporting formats across accredited institutions	Large-scale sequencing capacity supported by commercial and academic laboratories, with emerging national quality-control and accreditation frameworks.	Centralised, quality-controlled national network using approved assays with mandatory standards
**Sequencing modality**	Tissue and liquid CGP + multi-omics (SCRUM-MONSTAR)	Panel NGS	Panel NGS/WES	CGP/WGS/WTS (research)|
**Molecular tumour boards (MTBs)**	Mandatory, highly standardised multidisciplinary MTBs linked to reimbursement	Widely adopted at institutional or MTB group level; formats, documentation, and authority vary between hospitals	Highly variable MTB models, ranging from academic institutional boards to commercially facilitated services, with ongoing efforts toward standardisation.	Operated by metropolitan research-intensive cancer centres.
**Access to matched therapies and alignment with CGP**	Broad access to approved drugs, but persistent mismatch due to indication-based reimbursement and limited off-label use	Rapid regulatory approval of targeted agents, but delays in insurance coverage increase the financial burden on cancer patients	Access to targeted therapies expanding through the National Reimbursement Drug List, but provincial variation in reimbursement and co-payments persists.	“Right test, wrong access” Broad access to approved drugs through the PBS, but persistent mismatch between genomic testing results and treatment access due to indication-based reimbursement that limits biomarker-driven off-label use
**Representative initiative**	SCRUM-Japan/NCCH1901	K-MASTER/KOSMOS-I/II	National genomic platforms	PrOSPeCT/ASPiRATION/SUPER-NEXT
**Research-integrated precision oncology and trial access**	Nationwide genomic screening using multi-omics profiling linked to trial matching and regulatory-grade longitudinal real-world data (SCRUM-MONSTAR; conducted outside routine insurance coverage), alongside PPHS-based basket trials such as NCCH1901/BELIEVE that are linked to reimbursed clinical care, collectively enabling access to matched therapies for molecularly defined rare cancers.	Strong research capacity through hospital-based platforms and large-scale national initiatives: K-MASTER (2017–2021; ∼8000 patients, 55 institutions), KOSMOS-I pilot (2021–2022; 51% MGT rate, 29 sites via virtual cMTB), and ongoing KOSMOS-II (31 centers with central MTB and clinico-genomic database); strength in immuno-oncology and early-phase studies	Rapidly expanding clinical trial ecosystem leveraging large patient volumes and industry engagement, with increasing linkage to genomic screening.	Research-based programs utilising molecular profiling, linking genomic data to clinical decision-making, and integrating genomic testing with translational research demonstrate Australia's capacity for innovative precision oncology models, though these remain isolated examples rather than systematic national practice.
**Real-world data infrastructure, governance, and interoperability**	Centralised national genomic–clinical repository (C-CAT), complemented by regulatory-grade registries (e.g., SCRUM-Japan Registry); limited interoperability with other national health datasets	K-CURE project linking national cancer registry with clinical and genomic outcomes; national cancer clinical and genomic (CURE-NGS) database launched in 2025 covering ∼67% of cases nationwide	National clinical–genomic data platforms under development, operating within strict data-protection laws and increasingly adopting federated approaches.	Limited to research setting
**Workforce capacity**	Shortage of specialised genomic professionals; reliance on central expertise and virtual MTBs	Shortage of specialised genomic professionals; virtual MTBs and telemedicine not yet widely adopted in routine clinical practice	Shortages of specialised genomic professionals outside major urban centres, partially mitigated by national training initiatives and AI-assisted tools.	Shortage of specialised expertise in genomics; reliance on central expertise and virtual MTBs
**Major equity challenges**	Late-line CGP restriction, regional access	Out-of-pocket payment	Urban–rural disparities	Geographic remoteness
**Key policy levers**	C-CAT, standardised MTB	CURE-NGS, NHI expansion	NRDL expansion, provincial networks	National genomic alliances, telehealth

NHI, National Health Insurance.

## Methods

A non-systematic but structured narrative review with expert consultation was adopted to integrate heterogeneous sources of evidence, recognising that differences in health-system organisation, reimbursement mechanisms, regulatory environments, and research infrastructures across the four countries are not fully captured by conventional systematic review methodologies. Sex- and gender-specific analyses were beyond the scope of this review because the focus was health-system implementation rather than patient-level clinical outcomes.

First, a targeted review of documents and literature was conducted. National cancer control plans, genomic medicine policies, reimbursement guidelines, and regulatory frameworks were reviewed, including materials from the Ministries of Health in Japan, South Korea, and China, and from Australia's Department of Health and Aged Care. Peer-reviewed publications from oncology, genomics, and health-policy journals were examined to contextualise diagnostic capacity, uptake of molecular testing, precision oncology trial activity, and data governance structures. For Japan, particular attention was paid to literature describing nationally coordinated cancer genomic medicine initiatives and investigator-initiated precision oncology programs.^10^^,^^11^^,^^18^ For the other countries, we reviewed sources describing hospital-based implementation of next-generation sequencing in South Korea; large-scale commercial and academic sequencing ecosystems in China; and state- and federal-level approaches to delivering genomic sequencing as a clinical service, as well as research-embedded national genomics infrastructures in Australia.^16^^,^^19^

Second, expert consultation was conducted among the 19-member multidisciplinary author group, representing all four countries and selected based on country-specific expertise in precision oncology and related health-system implementation. Through iterative manuscript review and email or online discussions, experts validated and contextualised country-specific descriptions and identified implementation barriers. Feedback was synthesised by the lead authors, with differences resolved by consensus. Expert input contextualised published and policy evidence rather than serving as an independent source of quantitative data.

Finally, illustrative case examples were selectively incorporated to highlight representative implementation models and patient-pathway features, rather than to provide exhaustive programmatic descriptions. Review themes were synthesised iteratively using a hybrid approach that combined deductive framing informed by the Lancet Oncology Commission with inductive identification of cross-country patterns and divergences. This approach enabled structured regional comparison while preserving sensitivity to national context and implementation maturity.

Because the objective was to provide an implementation-oriented regional synthesis rather than an exhaustive systematic review, article selection and interpretation were informed by expert consultation and iterative consensus among the multidisciplinary author group.

## Search strategy and selection criteria

We conducted a structured narrative review with expert consultation. PubMed/MEDLINE was searched using predefined combinations of free-text terms and relevant MeSH terms related to precision oncology, cancer genomics, comprehensive genomic profiling, molecular tumour boards, genomic medicine, and real-world data, combined with country-specific terms for Japan, South Korea, China, and Australia. Governmental and policy documents were additionally identified from official national sources. Full search strategies, search dates, and inclusion and exclusion criteria are provided in the Supplementary Appendix 1. Because the objective was to provide an implementation-oriented synthesis rather than a systematic review, studies were selected based on relevance to national precision oncology implementation, health-system organisation, reimbursement, governance, and research integration.

## Regional landscape and patient pathway

Across Japan, South Korea, China, and Australia, precision oncology has evolved from a technology-driven concept to a health-system–embedded practice.^10^^,^^11^ However, the patient journey—from diagnosis to molecular testing, treatment selection, and follow-up—remains heterogeneous, shaped by differences in reimbursement, governance, research, and data infrastructure. These variations determine when patients access genomic testing and matched therapies and where bottlenecks arise (Fig. 1).

**Fig. 1:**
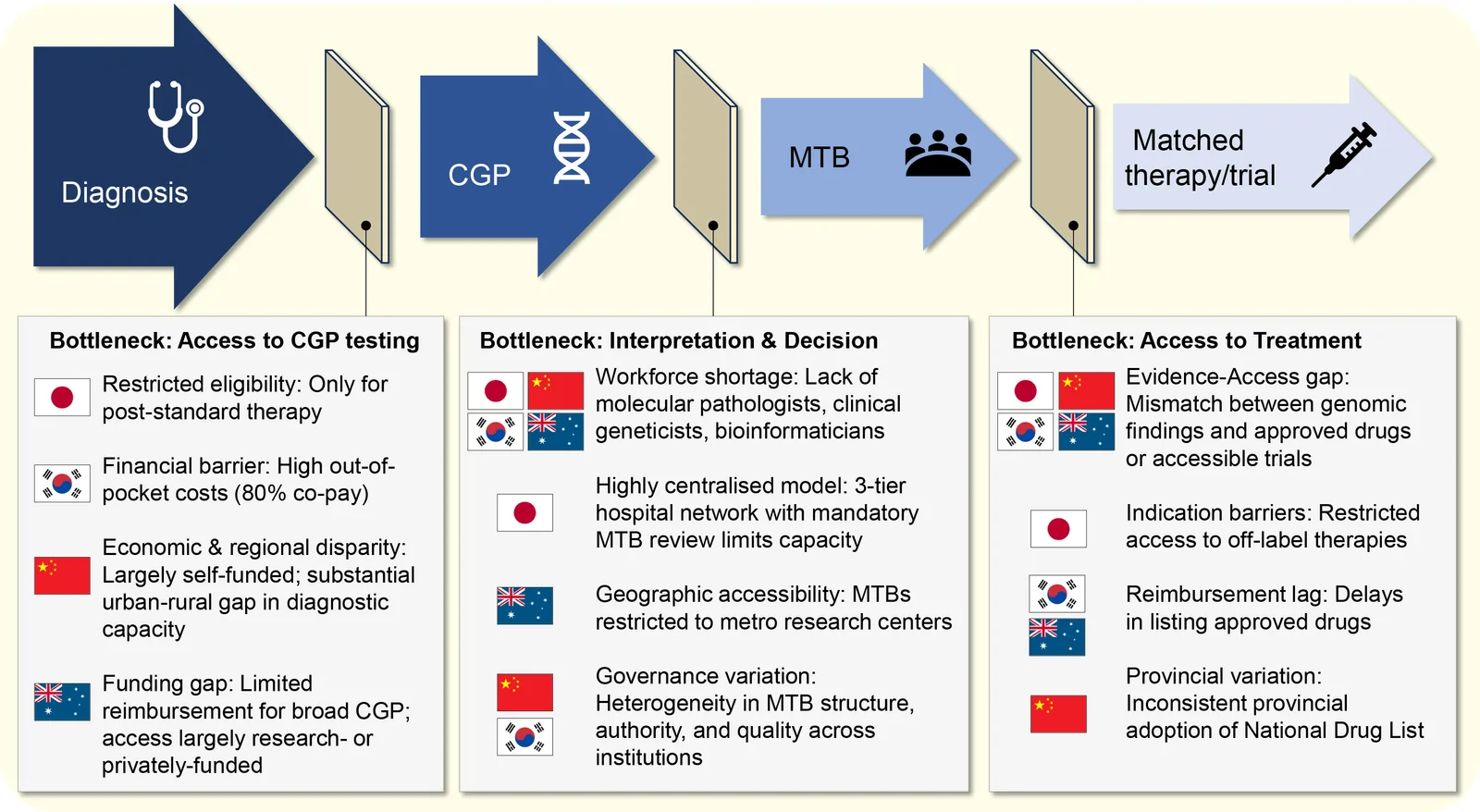
**Key bottlenecks in the Cancer Genomic Medicine Patient pathway.** This schematic outlines recurrent bottlenecks along the cancer genomic medicine pathway in Japan, South Korea, China, and Australia, from diagnosis to comprehensive genomic profiling (CGP), molecular tumour board (MTB) interpretation, and access to matched therapy or trials. Although sequencing capacity is established across the region, constraints arise at multiple stages, including eligibility restrictions and affordability of CGP, workforce and governance variability in MTBs, and misalignment between genomic findings and reimbursed treatment options. The figure emphasises that implementation barriers—rather than technological availability alone—shape real-world clinical impact.

Cross-country differences partly reflect distinct institutional trajectories. Japan developed a centrally coordinated national model, South Korea combined rapid NGS adoption with government-supported and hospital-based programmes, China expanded through a large public–private genomics ecosystem, and Australia developed a research-embedded model. These institutional contexts have shaped differences in standardisation, access, evidence generation, and integration into routine care.

### From suspicion to diagnostic work-up

In all four countries, the initial diagnostic phase is well established, with broad access to imaging, histopathology, and standard single-gene or small-panel biomarker testing. In Japan and South Korea, universal coverage and referral systems support timely evaluation, and guideline-recommended biomarkers such as *EGFR*, *ALK*, *HER2*, *RAS*, and *BRAF* are routinely available in tertiary centres.^20^, ^21^, ^22^ In China, diagnostic capacity varies geographically: urban centres provide comprehensive testing, while rural regions are expanding access through tele-pathology and tiered networks. In Australia, Medicare-funded services provide access to imaging and essential biomarkers, although patients in remote regions may face delays, and comprehensive multi-gene panels are not routinely available at diagnosis.

### Access to molecular testing and comprehensive genomic profiling

Comprehensive genomic profiling (CGP) represents a critical inflection point. Japan operates a nationally regulated, three-tier Cancer Genomic Medicine network (Core, Designated, and Cooperative Hospitals) with mandatory quality standards and molecular tumour board (MTB) review.^10^^,^^11^^,^^14^ While this ensures analytical robustness and centralised data submission, reimbursement is largely restricted to patients who have exhausted standard therapies, limiting earlier clinical utility.

In South Korea, next-generation sequencing capacity has expanded rapidly across tertiary hospitals and private laboratories. However, limited reimbursement means many CGP tests require substantial out-of-pocket payment, leading to socioeconomic disparities. The December 2023 policy revision, which increased patient co-payment rates from 50% to 80% for most cancers, further constrained access.^23^ China has achieved broad availability of CGP through a large commercial diagnostics market, particularly in urban centres. Ongoing standardisation efforts, including quality-control programs and pilot Laboratory Developed Test policies, aim to harmonise implementation, with increasing alignment through national accreditation frameworks. In Australia, key biomarkers are publicly reimbursed, and broader CGP panels are selectively funded through state programs and research initiatives; however, access varies by jurisdiction, and fee-for-service sequencing remains common.

### Molecular tumour boards and decision-making processes

Molecular tumour boards (MTBs) play an important role in interpreting genomic findings and facilitating treatment recommendations. However, the organisation and accessibility of MTBs vary substantially across countries. Detailed comparisons of MTB implementation and decision-making frameworks are discussed in the thematic section below.

### Access to targeted therapies and clinical integration

In Japan, targeted therapies are broadly available under universal insurance; however, indication-based reimbursement and restrictions on off-label use create a mismatch between CGP findings and treatment access. The BELIEVE/NCCH1901 basket trial, implemented under the Patient-Proposed Healthcare Services (PPHS) framework, provides genomically guided access to selected off-label agents and has enrolled over 290 patients across 18 cohorts.^18^ While NCCH1901 has expanded treatment opportunities, its scale remains insufficient to compensate for systemic constraints.

In South Korea, regulatory approval of targeted agents is relatively rapid, but delays in insurance coverage and substantial co-payments restrict uptake.^24^ Despite these reimbursement challenges, South Korea has built a robust precision oncology research ecosystem supported by nationwide genomic initiatives, virtual molecular tumour boards, and large-scale clinico-genomic databases, providing an important foundation for future implementation.^25^, ^26^, ^27^, ^28^ China has expanded access through the National Reimbursement Drug List, although provincial variation persists.^29^ In Australia, access is generally consistent through the Pharmaceutical Benefits Scheme (PBS); however, delays in listing newly approved agents can limit timely access.

### Follow-up, clinical trials, and data integration

Japan's outcomes infrastructure is supported by institution-based follow-up, the C-CAT centralised clinical–genomic repository,^11^ and PPHS frameworks enabling access to investigational therapies.^18^ The SCRUM-MONSTAR programme has emerged as an influential driver of evidence generation in molecularly defined rare cancers.^30^ Through integrated multiomics screening and a collaborative network, it has conducted 17 investigator-initiated trials evaluating therapeutic hypotheses across tumour types.^6^^,^^30^ The SCRUM-Japan Registry prospectively accumulates regulatory-grade real-world data with structured imaging intervals and auditable quality controls,^31^^,^^32^ and has contributed to regulatory approval using curated RWD as external control data.^31^^,^^33^ Importantly, the SCRUM-MONSTAR ecosystem has demonstrated measurable clinical impact beyond its role as a research platform. Patients who received genomically matched therapies had significantly longer overall survival than those receiving non-matched therapies (median overall survival, 19.1 vs 15.3 months; HR 0.767, 95% CI 0.710–0.829). These findings suggest that integrating comprehensive molecular profiling with trial access and multidisciplinary interpretation may improve patient outcomes by facilitating access to genomically matched therapies, highlighting implementation success beyond sequencing capacity alone.^30^

South Korea maintains a strong early-phase clinical trial ecosystem but lacks a unified national registry linking genomic data with long-term outcomes.^34^ China's clinical trial activity is expanding rapidly, supported by large patient volumes and emerging national data platforms integrating real-world evidence.^35^ Australia's research landscape is decentralised and lacks formal coordination, although initiatives linking genomic testing with trial matching and data sharing are expanding.

Cross-country comparison of implementation outcomes is limited by heterogeneity in testing eligibility, definitions, and outcome reporting. Available evidence and key gaps across four core implementation metrics—CGP access, access to genomically matched therapy, clinical outcomes, and economic evidence—are summarised in Table 2. Comparable national-level data remain limited, highlighting the need for standardised metrics for future regional benchmarking.

**Table 2:** Comparative framework for precision oncology implementation outcomes across Japan, South Korea, China, and Australia.

Core metric	Japan	South Korea	China	Australia
**Access to CGP**	Nationally reimbursed, but mainly after exhaustion of standard therapies	NGS widely available; high patient co-payment limits access	CGP widely available, particularly in urban centres, but largely self-funded	Broad CGP mainly available through research and selected programmes
**Access to genomically matched therapy**	Access to approved therapies is broad, but off-label access remains limited; research programmes provide additional pathways	**51% received molecularly guided therapy in KOSMOS-I**^a^	Expanding through clinical trials and NRDL-listed therapies; no comparable national matching rate identified	Research programmes link profiling to trial matching; no comparable national matching rate identified
**Clinical outcome evidence**	**SCRUM-MONSTAR: median OS 19.1 vs 15.3 months for matched vs non-matched therapy (HR 0.767)**^a^	No directly comparable survival estimate identified	No directly comparable survival estimate identified	No directly comparable survival estimate identified
**Economic evidence/cost-effectiveness**	Limited comparative evidence	Limited comparative evidence	Limited comparative evidence	Limited comparative evidence

a Programme-level evidence; estimates should not be interpreted as direct country-level comparisons.

## Thematic sections

### Diagnostics and testing infrastructure

Across Japan, South Korea, China, and Australia, next-generation sequencing capacity is well established; however, diagnostic infrastructures differ in organisation, quality assurance, and geographic accessibility. These differences shape both test reliability and integration into clinical care.

Japan's diagnostic infrastructure is characterised by a centralised model under the national Cancer Genomic Medicine programme. CGP is restricted to reimbursed assays within a three-tier network of Core, Designated, and Cooperative Hospitals, each subject to national standards and training requirements.^10^^,^^11^ This framework ensures analytical robustness and standardised reporting, but limits flexibility and may create bottlenecks at high-volume centres.^13^ South Korea has adopted an institution-accreditation model, requiring certification for NGS-based testing. While ensuring baseline quality, it permits heterogeneity in panel design and reporting formats across institutions.^15^ National harmonisation efforts are ongoing but not yet fully realised. China has achieved the largest expansion in testing volume and geographic coverage, driven by commercial and academic laboratories, with inland provinces adopting national training and quality-control standards. Australia combines strong public-sector diagnostic infrastructure with research-led innovation. However, universal access to CGP remains limited.^19^

### Access and affordability of biomarker testing and matched therapies

Access to biomarker testing and matched therapies remains a key bottleneck. Across all four countries, technological capacity has outpaced reimbursement, creating gaps between molecular identification and treatment.

In Japan, national insurance ensures access to CGP for eligible patients, but eligibility is largely confined to late-line settings. In South Korea, while key companion diagnostics are reimbursed, broader CGP often requires substantial out-of-pocket payment, leading to socioeconomic disparities.^24^ China's access landscape is shaped by regional and economic heterogeneity. Although CGP is widely available, most tests are self-funded, and access to targeted therapies remains inconsistent across provinces. Clinical trials and access programmes often serve as primary routes to matched therapy. Australia provides relatively stable access to essential biomarkers and therapies through Medicare and the Pharmaceutical Benefits Scheme; however, CGP remains limited and often depends on state-based or research programs.

### Decision-making and molecular tumour boards availability

Molecular tumour boards (MTBs) are central to translating genomic data into clinical decisions, yet availability and governance vary widely. Japan operates one of the most standardised MTB systems globally, with MTBs convened at all Core and Designated Hospitals.^10^ South Korea has widely adopted institutional MTBs, though variation in structure and documentation persists, and MTBs typically function as advisory bodies.^15^^,^^36^ China exhibits diverse MTB models, ranging from academic boards to commercially facilitated services, with ongoing standardisation efforts.^37^

In addition to somatic alterations, CGP may identify variants suggestive of hereditary cancer predisposition. Approaches to germline findings remain heterogeneous and linked to MTB structures and access to genetic counselling. In Japan, secondary germline findings can be reported within a regulated framework, but confirmatory testing is not reimbursed, creating a gap between identification and diagnosis. In South Korea, genetic counselling capacity is expanding, but national certification and reimbursement remain limited.^38^ China shows greater variability, with confirmation largely dependent on local expertise and self-payment. Australia demonstrates more consistent integration of oncology and clinical genetics services, although access remains uneven. Overall, these differences highlight the need for clearer governance and workforce capacity for managing germline findings.

### Workforce capacity and training

Workforce capacity remains a critical constraint on precision oncology implementation. Japan and South Korea face shortages of molecular pathologists, clinical geneticists, genetic counsellors, and bioinformaticians, reflecting the rapid expansion of testing relative to training capacity.^15^ China is actively addressing its workforce challenges by integrating AI-assisted decision support systems and expanding specialised training programs across provincial hospitals.^39^ In Australia, national policy documentation highlights workforce issues such as shortages of genomic professionals and variation in training pathways, underscoring the need for more coordinated capability building.^40^ Notable efforts are underway to develop a competent genomics workforce in Australia.^41^^,^^42^

### Research initiatives and networks

The four countries have developed distinct genomic ecosystems shaped by policy, research integration, and regulation (Fig. 2). Japan's ecosystem is anchored by SCRUM-Japan, a nationwide genomic screening and trial-matching program enrolling over 40,000 patients. Its LC-SCRUM Asia and MONSTAR-SCREEN initiatives have identified rare variants and contributed to drug development.^6^^,^^30^ MONSTAR-SCREEN's integration of multi-omics profiling has enabled finer biological characterisation of tumours, supporting translational science and early-phase trial design.^30^

**Fig. 2:**
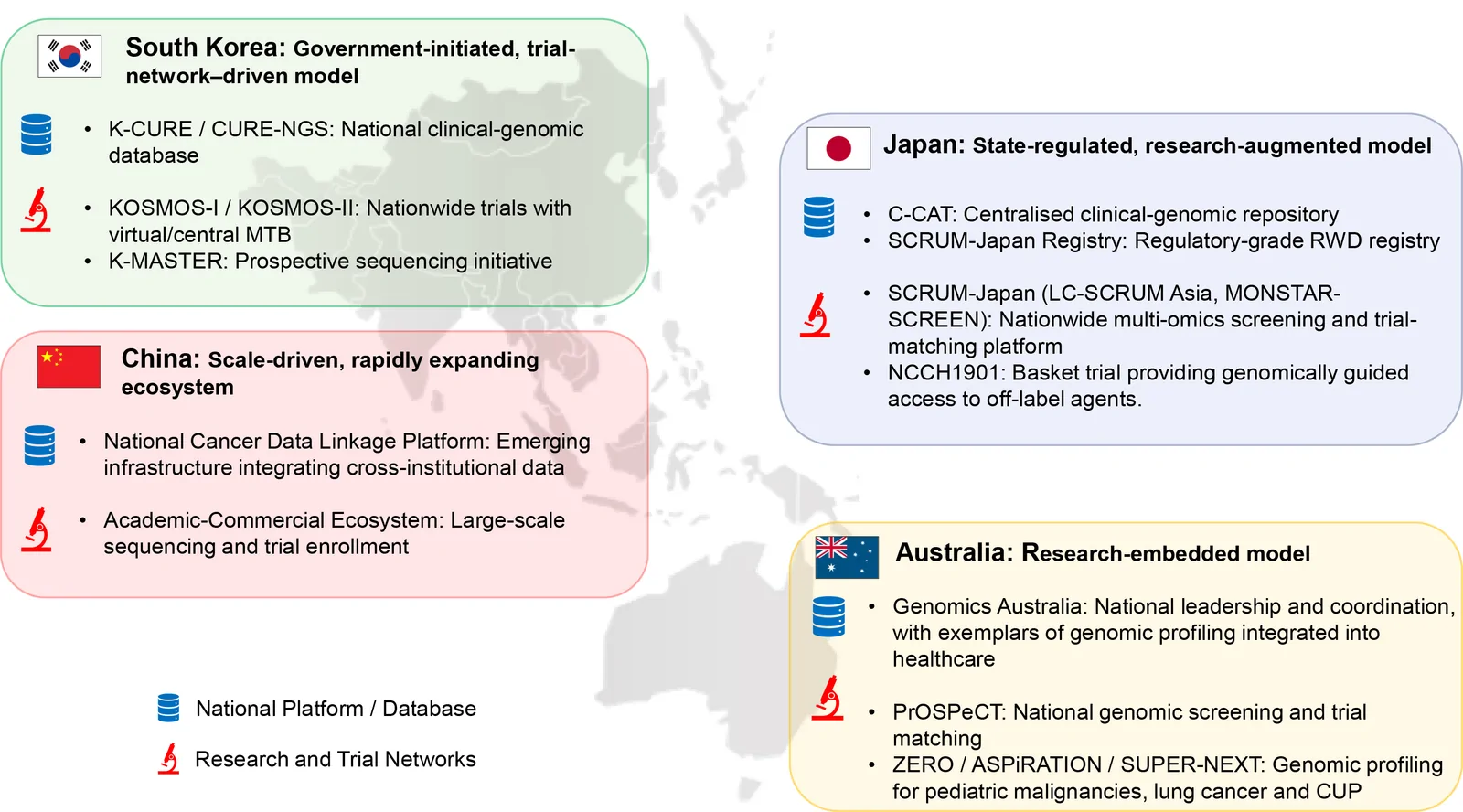
**Research and national genomics initiatives.** This figure summarises the dominant organisational models supporting cancer genomics research and implementation in Japan, South Korea, China, and Australia. Japan combines a state-regulated clinical genomics framework with research-integrated screening and real-world data platforms. South Korea has developed government-initiated, trial-network–driven programmes linking sequencing with central or virtual MTBs. China demonstrates a scale-driven ecosystem supported by large sequencing capacity and emerging national data platforms. Australia operates a research-embedded model integrating genomics into healthcare across jurisdictions. Together, these models illustrate distinct but complementary approaches shaped by governance structures and health-system design.

South Korea also maintains strong research capacity through hospital-based platforms and large-scale national initiatives. The K-MASTER program (2017–2021) established a government-supported precision medicine framework, enrolling approximately 8000 patients across 55 institutions during its initial prospective cohort, which laid the foundation for subsequent nationwide precision oncology initiatives.^25^^,^^26^ Building on this foundation, the KOSMOS-I pilot study (2021–2022) demonstrated feasibility of a nationwide virtual cMTB, achieving molecularly guided therapy in 51% of enrolled patients across 29 sites.^27^ This high treatment-matching rate highlights the potential of coordinated national genomic networks to translate molecular profiling into actionable therapeutic decisions at scale. The subsequent KOSMOS-II trial has further expanded this platform to 31 centers, operating a central molecular tumour board to match patients with targeted therapies and develop a clinico-genomic database.^28^

China has developed one of the world's largest genomics research ecosystems. Commercial and academic laboratories produce vast genomic datasets, and national initiatives support population-scale sequencing. Research integration is being enhanced through implementation of unified data standards and cross-provincial interoperability frameworks. These strategic efforts are systematically addressing previous challenges in data fragmentation and governance variation.^43^

Australia has an active research ecosystem in precision oncology. Examples of research-embedded initiatives in cancer include the PrOSPeCT program, which provides molecular profiling and trial-matching for patients with advanced cancers; the ASPiRATION study, a multicentre prospective cohort study linking comprehensive genomic data to clinical decision-making in metastatic lung cancer^44^; and the SUPER-NEXT program, a national initiative applying whole-genome and transcriptome sequencing (WGTS) to cancers of unknown primary (CUP).^45^ A major national investment in genomics, Australian Genomics was a collaborative partnership spanning more than 80 organisations, aimed at accelerating the integration of genomics into healthcare across multiple disease areas, not limited to cancer.^46^

### Data governance, ethics, and legal frameworks

Japan's C-CAT platform exemplifies a centralised data governance model, although long-term follow-up integration and interoperability remain limited.^11^ Japan also enacted the Act on the Promotion of Genome Medicine in 2023, which promotes research and clinical application of genome-based medicine with emphasis on the importance of protection of individuals' genetic information and privacy. South Korea launched a new project in 2025 to build a national cancer clinical and genomic (NGS) database, covering approximately 67% of cases nationwide.

China's data governance is shaped by strict regulatory frameworks—including the Personal Information Protection Law (PIPL) and Data Security Law— which impose stringent controls on data sharing and cross-border transfer. These regulations provide an essential and secure legal foundation, ensuring that the development of a unified national genomic ecosystem is conducted within a robust framework of privacy and biological sovereignty.^47^

Australia has established a national framework to guide the integration of genomics into healthcare.^40^ The newly formed national agency, Genomics Australia, will set a broad national strategy for embedding genomics in healthcare beyond cancer. The agency provides leadership, coordination, and expert guidance to support the equitable, effective, and ethical implementation of genomic technologies in routine clinical practice. In collaboration with all Australian governments, Genomics Australia is finalising the National Health Genomics Policy Framework and Implementation Plan 2026–2030, which will serve as the country's official genomics strategy, defining strategic priorities and providing a roadmap for nationwide implementation across jurisdictions.

### Equity and inclusion

Equity challenges cut across all thematic areas. In Japan, age, geography, and institutional capacity influence access to CGP. In South Korea, out-of-pocket costs exacerbate socioeconomic disparities. China faces profound urban–rural gaps in diagnostic access and affordability. In Australia, equity is prioritised in national frameworks and major policies,^41^ with attention to Aboriginal and Torres Strait Islander peoples and culturally and linguistically diverse communities. However, without deliberate implementation, precision oncology may amplify existing disparities. Importantly, these inequities arise from both structural barriers—including reimbursement policy, workforce distribution, health-system organisation, and digital infrastructure—and individual-level factors such as socioeconomic status, geographic location, and health literacy. Addressing these disparities will require coordinated policy interventions targeting both health-system design and patient-level access. These observations suggest that equity should not be viewed solely as a patient-level issue but rather as a systems-level property shaped by governance, reimbursement policy, workforce capacity, and data infrastructure. Consequently, reducing disparities requires coordinated reforms across the entire precision oncology ecosystem rather than isolated interventions targeting individual patients or institutions.

## Barriers and enablers

Despite substantial advances in cancer genomics and precision oncology across Japan, South Korea, China, and Australia, systemic, financial, regulatory, and workforce-related barriers continue to constrain equitable implementation. At the same time, each country has developed enabling structures that provide a foundation for future progress and regional collaboration.

### Systemic and financial barriers

A central challenge lies in the misalignment between diagnostic capacity and treatment access. In Japan, CGP is largely restricted to late-line settings, limiting the clinical value of identifying actionable alterations earlier in the disease course. Although universal health coverage provides strong financial protection, delays in reimbursement for newly approved targeted therapies and limited pathways for off-label use create persistent gaps between molecular findings and therapeutic action.^18^ Operational burdens associated with MTBs and data submission further contribute to variable turnaround times and regional capacity constraints.^13^^,^^48^

In South Korea, financial barriers are more pronounced. While key companion diagnostics are reimbursed, broad CGP panels often require substantial out-of-pocket payment, resulting in socioeconomic disparities and institution-dependent testing strategies. Rapid regulatory approval of targeted agents is not consistently matched by timely reimbursement, constraining real-world uptake. Nevertheless, South Korea has simultaneously developed nationally coordinated precision oncology programs that demonstrate strong implementation capacity beyond reimbursement policy. China faces the widest disparities: although CGP and targeted therapies are widely available in major urban centres, affordability and uneven provincial insurance coverage limit access in rural and less-resourced regions. Australia provides relatively equitable public coverage for essential biomarkers and many targeted therapies, yet not broad CGP routinely.

### Regulatory and data governance barriers

Regulatory and data governance frameworks represent another major barrier. Japan's centralised data infrastructure supports standardised genomic data collection, yet integration of long-term outcomes and interoperability with other national health datasets remain limited.^11^ South Korea initiated a national project in 2025 to establish a cancer clinical and genomic (NGS) database, enabling secure, de-identified data sharing for research.

China's regulatory environment prioritises data protection through stringent laws governing personal information and data security. These frameworks establish a secure and regulated environment that is supporting the adoption of collaborative models, such as federated learning, to facilitate data integration within a protected national ecosystem. Australia's regulatory framework for genomic testing is managed through the Therapeutic Goods Administration (TGA)'s medical device section as “in vitro diagnostics”. However, exemption is given for laboratory developed testing in a research setting. The regulation of funded testing (both public and private) is managed through the requirement for providers to be accredited by National Association of Testing Authorities (NATA) for compliance with the National Pathology Accreditation Advisory Council (NPAAC) standards. The accreditation framework includes inter-laboratory quality assurance testing but there is no national registry related to utilisation and outcome monitoring.

Another important area for regulation is prohibition of genetic discrimination. The four countries have taken diverse approaches to this area. Japan and China do not have legally binding regulation although Japan's Act for the Promotion of Genome Medicine has a description of the need of prevention of genetic discrimination. South Korea's law, the Bioethics and Safety Act, explicitly prohibits discrimination based on genetic information, while Australia enacted a new legislation which bans the use of genetic information in life insurance in 2026.

### Workforce and capacity barriers

Workforce shortages constitute a shared barrier across the region. Japan and South Korea face deficits in molecular pathologists, clinical geneticists, genetic counsellors, and bioinformaticians, reflecting the rapid expansion of genomic testing relative to training capacity. China's workforce challenges are being addressed by prioritising the dissemination of advanced expertise from urban centers to provincial hospitals through structured medical consortium networks.

### Key enablers across the region

Despite these barriers, several enabling factors provide a strong foundation for accelerated progress. Centralised quality frameworks, such as nationally coordinated genomic medicine programs, enhance test reliability and standardisation. Research-integrated models that link genomic screening with clinical trials have proven effective in expanding patient access to matched therapies while generating policy-relevant evidence. Large-scale sequencing capacity and active biotechnology sectors, particularly in China, support innovation and rapid technology diffusion. Federated and collaborative data initiatives, exemplified by national genomics programs and research networks in Australia and Japan, demonstrate feasible pathways for balancing data sharing with privacy protection.

Importantly, these enablers are not confined to individual countries. Regional reference laboratories, pooled procurement strategies, harmonised laboratory standards, and interoperable data frameworks represent transferable solutions that could mitigate common barriers across the Asia–Pacific region. Experience across the four countries suggests that nationally coordinated genomic medicine programmes, virtual molecular tumour boards, and public reimbursement of essential biomarker testing can reduce geographic and institutional disparities by improving access to specialist expertise and standardised diagnostic pathways. Leveraging these shared strengths through cross-country collaboration offers a pragmatic route to improving equity, efficiency, and sustainability in precision oncology implementation.

## Policy and implementation implications

Based on their expected impact and feasibility, seven priority actions are proposed, building where possible on existing regional models such as Japan's nationally coordinated genomic medicine network, South Korea's virtual MTB initiatives, China's provincial networks, and Australia's research-integrated genomics programmes. Immediate priorities emphasise adaptation of existing infrastructure, whereas resource-intensive expansion of workforce, data systems, and reimbursement is positioned as a medium-term priority.

### Expand timely access to comprehensive genomic profiling (CGP)

Restrictive eligibility and incomplete reimbursement limit the clinical impact of CGP. In the short term, ministries and payers should broaden eligibility beyond late-line settings and reduce out-of-pocket costs, particularly for patients with advanced disease or rare cancers. In the medium term, policies enabling earlier-line testing, repeat profiling to capture acquired resistance, and tumour-agnostic indications would maximise clinical utility while supporting learning health systems. Expanding access should be treated as a high-priority policy action.

### Strengthen and harmonise molecular tumour board (MTB) systems across the region

MTBs are central to translating genomic results into clinical decisions, yet governance and capacity vary widely. Immediate actions should prioritise routine funding, standardised documentation and reporting, and minimum expertise requirements. South Korea's KOSMOS-I virtual cMTB provides a regional example of how shared expertise can extend genomic decision support across institutions.

### Improve timely access to targeted therapies through streamlined reimbursement and adaptive health technology assessment (HTA) approaches

Reimbursement delays and restrictive indications constrain access. Short-term measures such as conditional reimbursement, managed access programs, and coverage with evidence development could reduce time-to-access. In the medium term, aligning evidentiary requirements for biomarker–drug pairs across countries would streamline adoption. Future reimbursement reforms should also consider long-term sustainability, including cost-effectiveness and budget impact, to ensure equitable and scalable implementation of precision oncology within publicly funded health systems.

### Build and sustain workforce capacity in cancer genomics

Workforce shortages in molecular pathology, genetics, bioinformatics, and genomic counselling remain a major bottleneck. Immediate actions include expanding funded training positions and supporting virtual expertise-sharing. Medium-term strategies should focus on regional training consortia, competency-based certification, and shared curricula.

### Establish interoperable data infrastructures using federated models

Fragmentation and limited interoperability constrain evidence generation. In the short term, harmonised data standards, metadata definitions, and quality benchmarks should be prioritised. Over the medium term, federated analytics frameworks enabling secure cross-border analysis offer a pragmatic solution while respecting national data-protection laws and supporting outcomes monitoring.

### Prioritise equity and inclusion across the patient pathway

Geographic, socioeconomic, and demographic disparities persist. Short-term interventions include subsidised testing, virtual MTBs, and decentralised sampling pathways.^49^ Medium-term policies should address paediatric and rare cancers and integrate community-based providers to reduce urban–rural gaps. Rare cancers and paediatric malignancies are particularly vulnerable to inequitable access because limited case volumes restrict the availability of companion diagnostics, specialist expertise, and genomically matched clinical trials. Research-integrated genomic programmes and virtual molecular tumour boards may help address these disparities by expanding access beyond major academic centres.

### Promote structured regional collaboration to accelerate innovation

The Asia–Pacific region has complementary strengths that can be leveraged through collaboration. Short-term opportunities include joint trials, shared variant-interpretation frameworks, and coordinated training. Over time, regional genomic reference datasets and aligned regulatory dialogue could position the region as a global leader.

Collectively, these actions provide a practical roadmap for strengthening precision oncology. Aligning national reforms with regional collaboration offers a feasible path toward more timely, equitable, and sustainable genomic medicine. Feasibility and resource requirements differ across these actions: virtual expertise-sharing and adaptation of existing networks may be implemented incrementally, whereas expansion of CGP reimbursement, workforce capacity, and interoperable data infrastructure requires greater sustained investment and formal evaluation of budget impact.

Expanding precision oncology involves trade-offs between equity, efficiency, affordability, and sustainability. Japan combines standardisation with restricted CGP eligibility; South Korea rapid adoption with substantial co-payments; China broad expansion with regional and affordability gaps; and Australia research-integrated implementation with limited routine CGP access. These contrasting approaches highlight the need to balance broader access with sustainable financing and implementation capacity.

## Future directions

Over the next decade, Japan, South Korea, China, and Australia are positioned to advance precision oncology through coordinated investment in diagnostics, data systems, and workforce capacity.

First, the maturation of multi-omics technologies—including whole-genome and transcriptome sequencing, proteomics, and circulating tumour DNA–based minimal residual disease assays—will enable earlier and more precise patient stratification.^50^ As these platforms move into clinical practice, they may shift precision oncology upstream, supporting earlier intervention and adaptive treatment strategies. Countries with strong research-integrated infrastructures will be well placed to generate clinical utility evidence and inform reimbursement reform.

Second, artificial intelligence is expected to enhance genomic interpretation and clinical decision-making. A national evaluation in Japan demonstrated higher concordance between AI-assisted treatment recommendations and expert consensus than conventional MTB processes.^51^ As workforce constraints intensify, AI-enabled interpretation and digital pathology will likely support standardised decision-making and expand access to expertise across diverse settings.^52^

Third, the timing of genomic testing may evolve. Evidence suggests that first-line CGP in untreated metastatic solid tumours can identify actionable alterations and facilitate matched treatment.^53^ These findings support reconsideration of late-line testing policies and highlight the value of earlier genomic integration.

Fourth, national genomic data infrastructures are expected to mature through centralised and federated models enabling interoperability, longitudinal outcomes tracking, and learning health systems. Shared standards and secure analytic frameworks will be critical for accelerating evidence generation while respecting data-protection requirements.

Progress will depend on equitable access to high-quality genomic testing, timely linkage to effective therapies, a digitally enabled workforce, and interoperable data ecosystems supported by strengthened regional collaboration.

## Limitations

This narrative review has several important limitations. First, the availability, quality, and granularity of publicly accessible data vary across Japan, South Korea, China, and Australia. Differences in health-system transparency, reporting practices, and regulatory requirements may result in uneven representation of national capabilities and challenges. Second, precision oncology is a rapidly evolving field; ongoing changes in reimbursement policies, diagnostic technologies, regulatory frameworks, and national strategies may alter aspects of the landscape after writing.

Third, although expert consultations were incorporated to contextualise policy environments and real-world implementation barriers where possible, reliance on published literature and publicly available policy documents may not fully capture the nuances of institutional practices, stakeholder perspectives, or on-the-ground implementation challenges that experts could have illuminated. Fourth, substantial intra-country heterogeneity—such as urban–rural disparities in China, institutional variability in South Korea, and regional differences in Australia and Japan—limits the generalisability of national-level descriptions. Fifth, cross-country comparisons are constrained by differences in terminology, regulatory scope, and the maturity of genomic medicine infrastructures, which complicate direct benchmarking. Finally, because this review adopted a structured narrative approach with expert consultation rather than a systematic review methodology, selection bias and citation bias cannot be excluded. Although efforts were made to incorporate diverse literature and cross-country expertise, the synthesis may have been influenced by the authors' research experience and the availability of published evidence.

Despite these limitations, this review provides a structured, comparative synthesis that identifies consistent patterns, shared barriers, and actionable policy insights relevant to precision oncology implementation across the Asia–Pacific region.

## Conclusions

Japan, South Korea, China, and Australia have established national or large-scale precision oncology infrastructures integrating cancer genomics into clinical care, although implementation varies in scope and maturity. Differences in access to comprehensive genomic profiling, reimbursement for targeted therapies, data-governance capacity, and workforce availability contribute to uneven patient experiences across the region.

This review maps patient pathways and key system domains, highlighting shared bottlenecks, particularly misalignment between genomic testing and timely therapeutic access. Strengthening national precision oncology frameworks, expanding workforce capacity, and improving reimbursement alignment for biomarker–drug pairs are essential to achieving equitable implementation. The experience of SCRUM-MONSTAR illustrates that successful implementation of precision oncology should be evaluated not simply by sequencing capacity, but by its ability to increase access to genomically matched therapies and improve patient outcomes through integrated clinical and research infrastructures.

Equally important is the development of interoperable and secure data infrastructures that enable continuous learning and outcomes monitoring. Coordinated regional collaboration, leveraging complementary strengths across the four countries, offers a pragmatic pathway to accelerate innovation, reduce disparities, and build a sustainable precision oncology ecosystem in the Asia–Pacific region.

## Contributors

HB and TY conceived the review. HB and HO drafted the manuscript.

All authors contributed country-specific information, critically revised the manuscript, and approved the final version.

## Data sharing statement

As this is a narrative review, no original individual participant data were generated or analysed. HB and TY verified the literature and publicly available information underlying the review and were responsible for the decision to submit the manuscript.

## Editor note

The Lancet Group takes a neutral position with respect to territorial claims in published maps and institutional affiliations.

## Declaration of interests

HB reports honoraria from Ono Pharmaceutical, Taiho Pharmaceutical, and Guardant Health.

HO, YJC, JY, XH, NZ, JIK, KS, YS, and RY report no competing interests.

SMG reports research funding from the Australian National Health and Medical Research Council, the Australian Medical Research Future Fund, the US Department of Defense, the Victorian State Government, Illumina, and the National Breast Cancer Foundation (Australia); and honoraria from AstraZeneca.

SYK reports grants from the National Research Foundation of Korea, Gradient Bioconvergence, National Cancer Center Korea, GC Genome Corporation, and Osteoneurogen; royalties from CbsBioscience; consulting fees from AstraZeneca Korea and the Korean Academy of Medical Sciences; honoraria from the Korean Cancer Association, Korean Breast Cancer Society, Yonsei University College of Medicine, Gachon University, Boston Korea Project, Gwangju Institute of Science and Technology (GIST), and Samsung Medical Center; and patents from the National Cancer Center, Korea.

JYH reports consulting fees from AstraZeneca, Amgen, Takeda, Oncovix, Lantern Pharma, AbbVie, Novartis, Daiichi Sankyo, LG Chem, Daewoong, Bristol Myers Squibb, Roche, Ono, Pfizer, Boehringer Ingelheim, and Janssen; and honoraria from AstraZeneca, Merck, Janssen, Roche, Takeda, Yuhan, Pfizer, and Novartis.

MA reports consulting fees from GxD Inc.

TF reports honoraria from Amelieff, AstraZeneca, and MSD.

KK is a member of the Technical Advisory Group on Genomics (TAG-G) at the World Health Organization; KK reports no competing interests.

AJ reports consulting fees from the University of Glasgow.

T.Y. reports research funding from Taiho, Ono, Chugai, MSD, Bristol Myers Squibb, Caris MPI, Daiichi Sankyo, Eisai, Exact Sciences, FALCO Biosystems, Medical & Biological Laboratories, Merus N.V., Miyarisan Pharmaceutical, Natera, Nippon Boehringer Ingelheim, Pfizer, Sysmex, and Takeda; honoraria from Chugai, Merck Biopharma, Ono, and Takeda; and consulting fees from Sumitomo and Indivumed.
