# Supplementary material for: Landscape of cancer genomics and precision oncology in Japan, South Korea, China, and Australia
Source: Lancet Reg Health West Pac. 2026 Sep 10;74:101981. doi: 10.1016/j.lanwpc.2026.101981 (PMC13649522; doi:10.1016/j.lanwpc.2026.101981)
Supplement: Supplementary file 1 [file mmc1.docx]

**Landscape of cancer genomics and precision oncology in Japan, South Korea, China, and Australia**

**Appendix 1. Detailed literature search strategy and selection criteria**

**1. Information sources**
Literature and policy information were identified from the following sources:

- PubMed/MEDLINE
- Governmental and policy documents from Japan, South Korea, China, and Australia
- Reference lists of relevant publications and national policy documents

**2. Search period**
The search was conducted on August 16, 2026, and was restricted to publications available up to May 1, 2026.

**3. PubMed/MEDLINE search strategy**
The PubMed/MEDLINE search was conducted on August 16, 2026, covering publications from database inception through May 1, 2026. The search strategy was designed to identify literature addressing the implementation of precision oncology and cancer genomics in Japan, South Korea, China, and Australia. The following search string was used:

(

"precision oncology"[Title/Abstract]

OR "cancer genomics"[Title/Abstract]

OR "genomic medicine"[Title/Abstract]

OR "comprehensive genomic profiling"[Title/Abstract]

OR "molecular tumor board"[Title/Abstract]

OR "molecular tumour board"[Title/Abstract]

)

AND

(

implementation[Title/Abstract]

OR access[Title/Abstract]

OR reimbursement[Title/Abstract]

OR policy[Title/Abstract]

OR governance[Title/Abstract]

OR infrastructure[Title/Abstract]

OR "health system"[Title/Abstract]

OR "healthcare system"[Title/Abstract]

OR "real-world data"[Title/Abstract]

)

AND

(

Japan[Title/Abstract]

OR "South Korea"[Title/Abstract]

OR Korea[Title/Abstract]

OR China[Title/Abstract]

OR Australia[Title/Abstract]

)

Additional relevant publications were identified through reference lists of eligible articles and national policy documents. Because this was a structured narrative review rather than a systematic review, literature identified through the search was used to inform the thematic synthesis together with policy documents and expert consultation.

**4. Eligibility criteria**

**Inclusion criteria:**
Publications and documents were considered eligible if they addressed precision oncology implementation in Japan, South Korea, China, or Australia and provided information on one or more of the following domains:

- genomic testing and comprehensive genomic profiling
- molecular tumour boards and genomic interpretation
- access to genomically matched therapies
- reimbursement and health policy
- data governance and real-world data infrastructure
- workforce capacity
- equity and access
- national genomic medicine or precision oncology research initiatives

**Exclusion criteria:**

- basic or preclinical genomics without relevance to clinical or health-system implementation
- studies unrelated to cancer
- purely technical sequencing studies without relevance to clinical implementation or health-system organisation
- duplicate publications or superseded policy documents where a more current version was available

**5. Grey literature and policy documents**
Governmental and policy documents were identified through official websites of relevant ministries, national cancer agencies, regulatory authorities, and national genomic medicine programmes in each country. Relevant documents were selected according to the same implementation domains used for the peer-reviewed literature.
